# SDN Controller Placement in IoT Networks: An Optimized Submodularity-Based Approach

**DOI:** 10.3390/s19245474

**Published:** 2019-12-12

**Authors:** Anh Khoa Tran, Md. Jalil Piran, Chuan Pham

**Affiliations:** 1 Modeling Evolutionary Algorithms Simulation and Artificial Intelligence, Faculty of Electrical & Electronics Engineering, Ton Duc Thang University, Ho Chi Minh City 700000, Vietnam; trananhkhoa@tdtu.edu.vn; 2 Computer Science and Engineering Department, Sejong University, Seoul 05006, Korea; piran@sejong.ac.kr; 3 Synchromedia—École de Technologie Supérieure, Université du Québec, QC H3C1K3, Canada

**Keywords:** Internet of Things (IoT), software-defined networking (SDN), network function virtualization (NFV), SDN controller placement

## Abstract

Software-Defined Networking (SDN) has opened a promising and potential approach for future networks, which mostly requires the low-level configuration to implement different controls. With the high advantages of SDN by decomposing the network control plane from the data plane, SDN has become a crucial platform to implement Internet of Things (IoT) services. However, a static SDN controller placement cannot obtain an efficient solution in distributed and dynamic IoT networks. In this paper, we investigate an optimization framework under a well-known theory, namely submodularity optimization, to formulate and address different aspects of the controller placement problem in a distributed network, specifically in an IoT scenario. Concretely, we develop a framework that deals with a series of controller placement problems from basic to complicated use cases. Corresponding to each use case, we provide discussion and a heuristic algorithm based on the submodularity concept. Finally, we present extensive simulations conducted on our framework. The simulation results show that our proposed algorithms can outperform considered baseline methods in terms of execution time, the number of controllers, and network latency.

## 1. Introduction

In a computer network, Software Defined Networking (SDN) [1,2] has become a major area of investigation in both academia and industry to enable a programmable network platform. The key technology in the SDN paradigm is the decomposition of the data plane and the control plane, in which network devices in the data plane perform the forwarding functions under the management and control of SDN controllers in the control plane. As a result, several SDN-enabled devices are equipped in current networks to provide flexible network architectures with minimizing capital expense (CAPEX) and operating expense (OPEX) [2].

**Figure 1:**
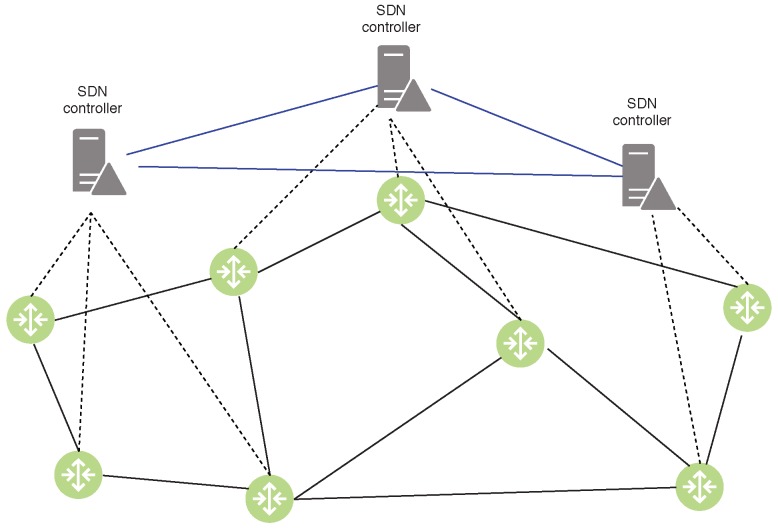
An example of the controller placement in the SDN network. The control plane is comprised of three controllers that can manage the whole network.

As the future network technologies, SDN and Network Function Virtualization (NFV) [1,3,4] are key technologies to bring smart services to Internet of Things networks [5]. As a result, several network architectures are now managed under the centralized manner control plane as a “brain" of the network and network functions are flexibly virtualized to deploy distributively by virtual network functions (VNF). However, there still exist many challenges to place an efficient control plane in the network. The authors of [6] firstly illustrated the solution to place a single controller to cover the entire network, but this architecture implies several issues to satisfy practical issues, such as reliability and resilience, especially in the case of distributed networks such as IoT networks. In such networks, edge computing is invoked to deploy edge nodes along the edge of networks in order to reduce the latency of services. A single controller has difficulty controlling every node of networks with real-time response. Furthermore, services in edge networks are often referred to different service providers under various protocols. Each controller is responsible for specific domains of a specific network provider. Such scenarios question them to find the efficient number and location of controllers for their networks. Despite attracting several studies, this topic is still open until now because of the growth of IoT networks. To briefly recall this concept, we can summarize the controller placement problem as follows. Given a set of nodes (i.e., SDN-enabled nodes) in an IoT network, the objective is to look for: (i) an efficient set of controllers to control the entire network; and (ii) a set of locations to place them in order to satisfy some specific constraints [6]. An example of the controller placement problem in SDN network is illustrated in Figure 1.

To propose a new solution for this problem, we advocate the submodular theory [7]: (i) to formulate the controller placement problem with different use cases that happen in practice; and (ii) to design efficient algorithms to solve them. In the state of the art, submodularity optimization is explained in different studies of graphs, combination optimization, etc. Recently, submodular functions get much attention in research as a promising tool to deal with Non-deterministic Polynomial-time hard (NP) problems, such as sensor placement and social networks. Inspired by them, we observe that the submodular theory perspective can uncover issues of the controller placement problem in IoT networks. In general, given a set of locations where controllers can be placed (which can be represented as a network graph), the goal is to find a set of controllers such that this set can maximize a predefined “submodular" set function.

Unlike the prior works in this field that propose different algorithms for different optimization formulations, we instead develop a universal framework under the submodular theory to apply in various use cases, from the basic to the complicated problems. Furthermore, the submodularity method enables a flexibly designed objective function to construct the optimization problem. Mathematically, we illustrate that the submodularity optimization is the efficient mechanism for the SDN controller placement problem in IoT network in terms of ease of formulation, simple implementation, and low complexity.

We summarize the major contributions of this paper with the following aspects:

- Our work applies the submodular theory to build an optimization framework for solving the class of controller placement problems. Although the submodularity method is utilized in many fields (e.g., sensor placement, wireless communication, and wi-fi network), to the best of our knowledge, this theory is not exploited well in the field of the controller placement problem.
- By formulating the controller placement problem in different use cases, we propose corresponding algorithms to find near-optimal solutions based on the covered set concept of submodularity. Although this problem invokes various forms, mathematically, we show that they are different instances of the basic submodular formulation.
- To evaluate the system, we provide an extensive simulation in which all introduced use cases are conducted. Compared to baseline methods, our proposed algorithms can obtain a promising performance in terms of execution time, network latency in the control channel, and the number of placed controllers in the network.

The rest of this paper is organized as follows. In Section 2, we discuss the related works on this topic. We next study different use cases of the controller placement problem and formulate the optimization based on submodularity in Section 3. Corresponding to each problem in this section, we also present the designed algorithms. We investigate a numerical result to evaluate our proposed methods in Section 4. Finally, we conclude the paper in Section 5.

## 2. Related Work

There are several advantages of SDN-based architecture that attract much attention in practice and research. The key point in the SDN networks is the decomposition of the control plane from the data plane; such technology enables efficient control and management mechanisms in networks. However, the dependency of the data plane in SDN networks on controllers may reduce the reliability of networks as some wrong placements or failures often occur in the control plane. Therefore, improving network performance and reliability is a major issue explored in the state of the art. To review trends in this area, we discuss the main topics in this section.

### 2.1. Single Placement Scheme

In the first work introduced by Heller et al. [6], the main objective is to place a controller that can cover every node in the network to satisfy some specific constraints, such as a threshold of resilience, minimum latency in communication, and fault tolerance. There are some studies [2,8] that focus on this topic and propose an efficient solution. In [8], the authors proposed a heuristic method that selects the node with the largest number of neighbor nodes. However, its solution does not guarantee a minimum latency to control the whole system. In [2], a greedy algorithm is introduced named as Greedy Routing Tree algorithm. The proposed algorithm outperforms the methods proposed in [8], but it has a high complexity in a large-scale setting.

However, single controller placement cannot fit in several cases of real systems [4]. For example, with a large system, as shown in [9,10], nodes are distributed in many locations which might degrade the system performance since there is a high latency in the control channel. Once the failure happens in the controller, it also raises a critical issue to find another candidate as backup [4,9].

### 2.2. Multiple Placement Scheme

Therefore, multiple controller placement is employed in distributed networks, especially in the IoT scenario, to address such issues; however, the problem becomes more complicated than the single placement problem [4]. Multi-controller placement scheme becomes more necessary in reality as multi-network providers coexist in the IoT network. Each network provider has different network topologies and benefits to be considered in its control plane. One of the related models is presented in [11], where the authors proposed an optimal resource utilization model to place controllers. Taking into account the same problem, Dixit et al. [12] considered an elastic model for distributed SDN controllers, which is more interesting and complicated. Some other authors consider this problem under a joint optimization problem (see, e.g., [4,9]).

As the new prior work, which is on the same topic and close to our study, the authors of [13] focused on placing SDN controllers in edge networks under a mixed integer programming problem to minimize the placement cost. They also considered the high complexity of the placement problem, which motivated them to use approximation methods to relax their problem into a tractable form in order to solve it using standard solvers and the supermodular method. However, the authors only considered a standard form of submodularity, which needs to be considered carefully to cover extensive cases in reality. For instance, some controllers in the real network are predefined by an administrator because of physical deployment reasons. There are only some flexible nodes that can scale up/down depending on user workloads. In such a case, the standard problem of submodularity will attempt to find an optimal subset of nodes that may not contain pre-defined nodes. This use case and further possible scenarios in SDN controller placements cannot be simply considered [13]. Therefore, in this work, we propose a robust framework to address varying use cases of this problem.

Among several prior works, controller placement problems are often formulated under Mixed Integer Linear Programming (MILP) [4]. Relying on some powerful solvers [13,14,15], such as Gurobi [16] and CPLEX [17], is a common way to find the solution. However, the time complexity is still a big issue in this field when considering a large-scale network. With a huge combination of nodes and links in the network, it may take several hours to achieve an optimal solution by these solvers; in some cases, solvers have some unknown errors due to over settings for their capacity. Therefore, this topic is still promising for research to find efficient solutions in terms of low time complexity.

## 3. Optimizing Covered Submodular Functions for the Controller Placement Problems

### 3.1. Preliminaries

We first summarize the concept of maximization submodular set functions with the following definition.

**Definition** **1.** *Given any real-valued set-function $f:2^{V}\to R$ with a finite set, V is submodular if, for any two set A,B⊆V, we have f(A)+f(B)≥f(A∪B)+f(A∩B) [7].*

This condition in Definition 1 is equivalent to

$$\begin{array}{c} f(A\cup k)-f(A)\geq f(B\cup k)-f(B),\forall A,B\subseteq V,\mathrm{and}\;k\in V\;\mathrm{such}\;\mathrm{that}\;A\subseteq B\;\mathrm{and}\;k\notin B. \end{array}$$

This definition is called the definition with first-order differences [7]. Furthermore, as presented in [7], submodular functions are defined in a discrete concept, but their properties are more similar to convex function. Consider f(.) as a non-decreasing function; it means that f(A)≤f(B),∀A⊆B⊆V. This is a strong property of this mechanism compared to others in order to design flexible objective functions f(.). Hence, following Lovász et al. [7], we have an important proposition that is used in our work as follows.

**Proposition** **1.** *Denoting $E={⋃}_{i=1}^{n}S_{i}$ and a submodular function f(.) on the set of subsets of V, we have $\sum_{i=1}^{n}f\left(S_{i}\right)\geq f\left(E\right),\forall S_{i}\subseteq V,n\geq 1.$*

We now introduce the controller placement problem based on the formulation of a submodular function.

### 3.2. Use Case 1: Maximum Covered Submodular Set Problem

We consider a general model with physical network nodes [9] as a graph G={V,E} where a set of physical nodes V can be seen as the vertex set of the graph and the set E of edges is to present connections between pairs of nodes.

Problem $P_{1}$: Considering the submodular function f(.) on the set of subsets of V and the maximum number of controllers *k* that can be placed, we represent the basic controller placement problem under the maximization submodular problem to find a subset S⊆V to control all nodes in the network such that:

- |S|≤k; and
- f(S) is maximized.

Mathematically, Problem $P_{1}$ can be written as

(1) $$\begin{array}{c} P_{1}:\max\left\{f\left(S\right):|S|\leq k,S\subseteq V\right\}. \end{array}$$

**Figure 2:**
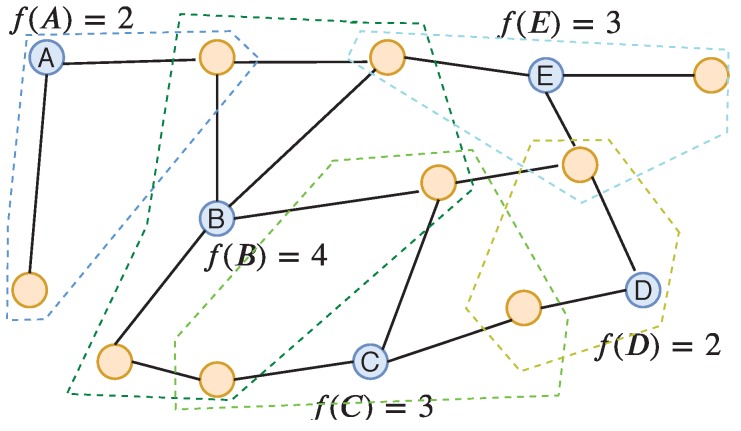
An example of submodular functions for SDN controller assignment: f(A)=2,f(A,B)=5,f(A,B,C)=7,f(A,B,C,D)=8,f(A,B,C,D,E)=9.

**Discussion and algorithm.** In this problem, f(.) can be formulated as a utility function when deploying the set *S* of controllers and *k* is the limited number of controllers that can be placed in the network. In general, given a non-decreasing submodular function f(.), $P_{1}$ can represent exactly the basic form of the controller placement problem [2] in the SDN network. A toy example in Figure 2 demonstrates submodular functions for SDN controller placement with a given 5 locations A, B, C, D, E to place controllers.

Based on Nemhauser et al. [18], when f(∅)=0, there exists a greedy incremental algorithm for $P_{1}$ with an approximation ratio $\frac{e}{e-1}$. For ease of notation, we denote $P_{1}$ as $P_{1}\left(f,V,k\right)$. To solve this problem, we can apply the Nemhauser’s algorithm [18]. The procedure of Nemhauser’s algorithm is very simple, as shown in Algorithm 1, in which the set of controllers is added one by one with the condition that each selected node must maximize the submodular function as $v=\arg\max_{v\in V}\left\{f\left(S\cup \left\{v\right\}\right)-f\left(S\right)\right\}$.

**Algorithm 1:** Nemhauser’s Algorithm for $P_{1}$.

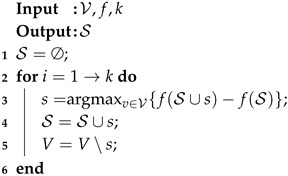



The formulation of $P_{1}$ can be easily applied in practice with a single or multiple controller(s) placement problem, where we can scale the given parameter *k*. In fact, the network system is often operated by a set of consistent controllers. Once the workload increases in some specific cases, the network provider needs to add more controllers to guarantee network performance. To deal with this problem, we further define the new problem of controller placement with given a set of consistent controllers and formulate under the submodular problem.

### 3.3. Use Case 2: Maximum Covered Submodular Set Problem with Given Requirement Set of Controllers

Problem $P_{2}$: Suppose that G=(V,E) is an undirected graph, and f(.) is a given submodular function on the set of subset of V. Given a limited number *k* of controllers that can be placed, and the given subset R⊆V of current controllers in the network, the control submodular problem is to find a subset S⊆V such that:

- The volume of S should be less than or equal *k*: |S|≤k;
- R is the subset of S: R⊆S; and
- f(S) is maximized.

Mathematically, Problem $P_{2}$ can be written as

(2) $$\begin{array}{c} P_{2}:\max\left\{f\left(S\right):|S|\leq k,R\subseteq S,S\subseteq V\right\}. \end{array}$$

**Discussion and algorithm.**$P_{2}$ is more specific than $P_{1}$ with a set S of controllers in the placement requirement. However, Algorithm 1 can approximate $P_{2}$. Similar to $P_{1}$, we denote $P_{2}$ as follows $P_{2}\left(f,V,k,R\right)$. $P_{2}$ can be transformed by reformulating the original function f(.) to $f^{\prime }\left(.\right)$ as the following equation

(3) $$\begin{array}{c} f^{\prime }\left(S\right)=\left\{\begin{array}{cc} f(S\cup \{R\}), & \mathrm{if}\;S\subseteq V\setminus \{R\},S\neq \{\emptyset \},\\ 0, & \mathrm{if}\;S=\emptyset . \end{array}\right. \end{array}$$

We can derive that $f^{\prime }\left(.\right)$ is a non-decreasing submodular function and $f^{\prime }\left(\emptyset \right)=0$. Hence, we can derive that the maximum value of $P_{2}\left(f,V,k,R\right)$ is equivalent to $P_{1}\left(f,V\setminus \left\{R\right\},k-|R|\right)$. By applying Algorithm 1, we can solve $P_{2}$ in an extended way. The intuition of the algorithm is to start from the given set R of controllers instead of the empty set as Algorithm 1 does. Hence, in the next step, the chosen node is selected to maximize the submodular value as shown on Line 3 of Algorithm 2.

**Algorithm 2:** Extended Nemhauser’s Algorithm for $P_{2}$.   **Input**: V,f,R,k   **Output**: S
**1** S={∅};**2** S← Apply Algorithm 1 on $P_{1}\left(f,V\left\{R\right\},k-|R|\right)$;**3** S=S∪R;

From both use cases, the submodular framework can formulate easily the controller placement problem with principle requirements. Significantly, it allows a flexible objective function to design the optimization problem. We next discuss extended problems in this area based on the submodular theory.

### 3.4. Use Case 3: Maximization Control Submodular Problem

We next widen $P_{2}$ by considering a connected graph of all controllers in the SDN control plane. A controller should be placed on a node that has the most connections to other nodes [2]. This observation can be seen as a simple intuition in placement strategies. Furthermore, all controllers should be connected to in their control channel. Based on that consideration, we formulate the Maximization Control Submodular problem as follows.

Problem $P_{3}$: Similar to $P_{2}$, we consider a connected graph G=(V,E), and a pre-defined submodular function *f* on the set of subsets of V, and given a limited number of controllers that can be placed. Then, we formulate the maximization control submodular problem that is to find a subset S⊆V such that:

- the sub-graph $G_{S}=\left(S,E_{S}\right)$ is connected with $E_{S}=\left\{\left(u,v\right)|\left(u,v\right)\in E,\forall u,v\in S\right\}$;
- |S|≤k; and
- f(S) is maximized.

Mathematically, Problem $P_{3}$ can be written as

(4) $$\begin{array}{c} P_{3}:\max\{f\left(S\right):|S|\leq k,G_{S}\;\mathrm{is}\;a\;\mathrm{connected}\;\mathrm{graph},S\subseteq V\}. \end{array}$$

**Discussion and algorithm.**$P_{3}$ is NP-hard since it can be formed as a covered set problem. Furthermore, it is clearly seen that $P_{3}$ can be formed as $P_{2}$ because *G* is a completed graph. Following Nemhauser et al. [18], $P_{2}$ is NP-complete so that $P_{3}$ is also NP-hard.

To find a solution for $P_{3}$, in this work, we propose Algorithm 3 under a search tree fashion. To do that, we denote $T_{v}$ as a subtree with the root node *v*, and STree(Z) as the tree that includes node *v* and all links connected between *v* and its neighbors. We design an iterative algorithm as shown in Algorithm 3. First, a specific node *v* is found to the satisfy conditions on Line 2. Then, all subtrees $T_{i}$ in a set TSet are included to cover $T_{v}$. When $T_{v}$ is covered, we remove $T_{v}$ and all adjacent edges to *v* from T. This adding step also ensures a connected graph at the end of the algorithm. Following steps in Algorithm 3, the algorithm aims to choose *v* such that each sub-tree $T_{i}$ with any children node of *v* has a size smaller than *k* but the size $|T_{v}|$ is greater than *k*. Furthermore, to reduce the number of sub-trees covered $T_{v}$, the algorithm does a grouping where each has a size that is smaller than *k* (Lines 9–10). Then, they will be merged into Z until its size is greater than *k* (Lines 6–11).

**Algorithm 3:** Search tree algorithm for $P_{3}$.

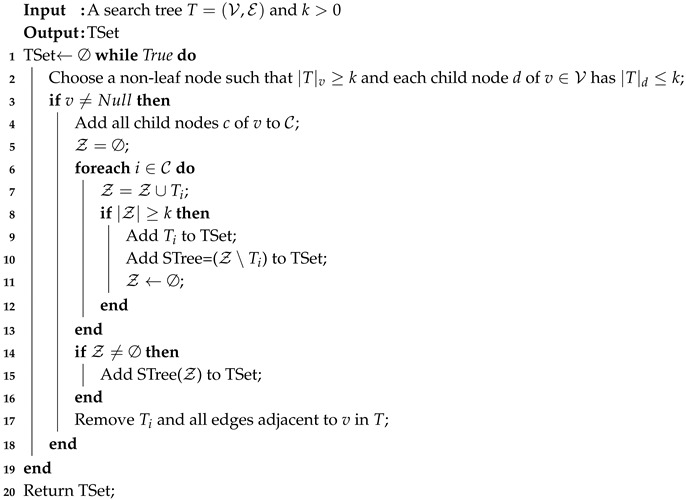



### 3.5. Use Case 4: Maximization Network Quality Factor Submodular Problem

We next consider another scenario with a set N of nodes that needs to be controlled, a set V of nodes to place controllers, and the communication rate *r*. For each location of *v*, we define a function f(v) to measure the connection from *n* to the controller at *v*. It means that $f\left(v\right)=\left\{n|d_{n,v}\geq r,\forall n\in N\right\}$, where $d_{n,v}$ is the communication rate between *n* and *v*. For a location subset S⊆V, we have $f\left(S\right)={⋃}_{s\in S}f\left(s\right)$. Now, given the limited number of *k* controllers that we can be deployed, the maximization control problem for a subset S can be presented as follows.

Problem $P_{4}$: Given a connected graph G=(V,E), and a pre-defined submodular function f(.) on the set of subsets of V, the maximization network quality factor submodular problem is to find a subset S⊆V such that:

- the sub-graph $G_{V,r}=(V,\left\{\left(n,v\right)|d\left(n,v\right)\geq r,\forall n\in N,v\in V\right\}$ is connected;
- the volume of S has been smaller or equal *k*: |S|≤k; and
- f(S) is maximized.

Mathematically, Problem $P_{4}$ can be written as

(5) $$\begin{array}{c} P_{4}:\max\{f\left(S\right):|S|\leq k,G_{V,r}\;\mathrm{is}\;a\;\mathrm{connected}\;\mathrm{graph},S\subseteq V\}. \end{array}$$

**Discussion**. By setting *r* large enough, we can transform $P_{4}$ to the familiar form of $P_{3}$. Therefore, $P_{4}$ is a special case of $P_{3}$ so that we can apply Algorithm 3 to find a solution for $P_{4}$. In fact, the formulation of $P_{4}$ is, in general, close to the control placement in practice with more aspects considered. First, in such a problem, the set of nodes that needs to be controlled and the set of locations of controllers are pre-defined as the real context. Second, in $P_{4}$, the formulation defines the network quality factor that makes more constraints as the real system to place controllers. Specifically, this factor can be considered as a number of hop count or latency measurements, which are often used in many prior works.

### 3.6. Use Case 5: Maximization Submodular Problem with Budget Constraints

We next discuss this problem from another point of view in which an economic aspect is invoked to constrain the placement decision. Specifically, it is a scenario with a network graph G=(V,E), a set N of nodes in the system that need to be controlled, and w(v),∀v∈V that indicates the deployment cost over v∈V. Given a budget *B*, we formulate the problem under the submodular formulation as follows.

Problem $P_{5}$: Given a connected graph G=(V,E), and a submodular function f(.) on the set of subsets of V, the maximization submodular problem with budget constraints is to find a subset S⊆V such that:

- S is a connected graph $G_{V,r}=(V,\left\{\left(n,v\right)|d\left(n,v\right)\geq r,\forall n\in N,v\in V\right\}$;
- the budget constraint $w\left(S\right)=\sum_{s\in S}w\left(s\right)\leq B$; and
- f(S) is maximized.

Mathematically, Problem $P_{5}$ can be written as

(6) $$\begin{array}{c} P_{5}:\max\left\{f\left(S\right):w\left(S\right)=\sum_{s\in S}w\left(s\right)\leq B,G_{V,r}\;\mathrm{is}\;a\;\mathrm{connected}\;\mathrm{graph},S\subseteq V\right\}. \end{array}$$

**Discussion and algorithm.** We can observe that $P_{5}$ can be an instance of $P_{4}$ in which the cardinality constraint is replaced by the constraint of the total weight of elements in the optimal set S. Therefore, we can apply Algorithm 3 for solving $P_{5}$ in which the cardinality constraint with the limitation of *k* elements is replaced by the budget constraint. With the same procedure as in Algorithm 3, Sviridenko [19] claimed that this is a $\frac{e}{e-1}$-approximation algorithm.

## 4. Numerical Results

In this section, we present the numerical results corresponding to five problems introduced in our work. Specifically, we focus on quantifying the result of algorithms proposed in this work and make a comparison with some baseline methods.

### 4.1. Settings

**Network settings**. We created our simulation environment with a physical network using Zipf’s law [20] to create random nodes and links in the network. Specifically, we generated a network topology with 200 nodes. To locate them, we first selected k=10 candidate nodes to form *k* clusters. For the instance of the IoT network, we represented these clusters as edge networks. Then, using Zipf’s law, we randomly assigned the remaining nodes into these clusters. After that, we randomly connected links between nodes in the graph until it became a connected graph. The propagation latency between a pair of nodes in the same cluster was set from 10 to 15 ms while connections between clusters were set from 20 to 30 ms; especially, 0 ms was set for unconnected pairs of nodes.

To evaluate the results of our proposed algorithms, we considered the following baselines:

- Optimal: We used the Gurobi solver [16] in Python to find the optimal solution. We considered this baseline to measure the optimality gap of our work in different settings. However, due to the limitation of Gurobi solver, the execution time was exponential when increasing the size of the network.
- Closest-distance: In this baseline, the placement mechanism was based on the distance between nodes [4]. First, we divided the network into clusters. Then, we placed controllers corresponding to cluster heads. This is one of the simplest methods to make a placement decision in the network without considering other aspects (e.g., budget).

### 4.2. Results

**Figure 3:**
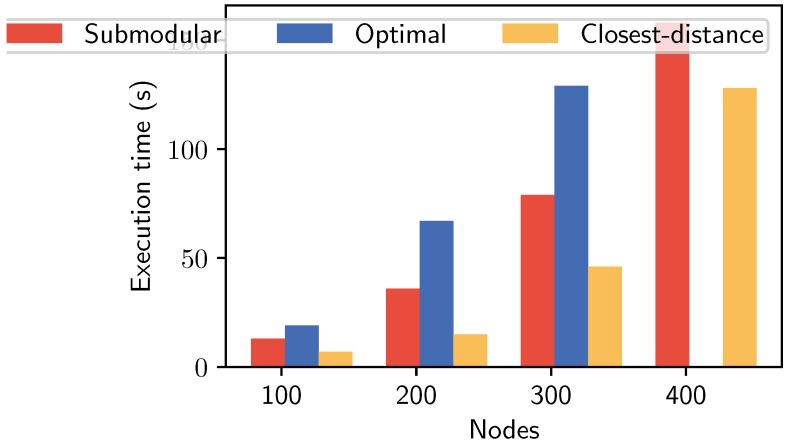
Execution time for different search spaces.

**Evaluation of execution time.** We first compared the execution time of our algorithms with other approaches, as shown in Figure 3. Accounting the results in three settings (i.e., the number of nodes was set from 100 to 300), we measured the execution in the Python environment with our station configuration (Intel(R) Dual Core TM i7-4790 CPU @ 3.6 GHz). The results show a good performance in finding a near-optimal solution with 13, 36, 79, and 158 s corresponding to 100, 200, 300, and 400 nodes, respectively. It reduced significantly the execution time compared to Optimal using Gurobi solver. Especially, with the large setting of 400 nodes, Gurobi solver could not obtain a result due to exceeding its limitation. In this comparison, Closest-distance has not only the lowest execution time, but also low performance in other aspects (we explain them in the next evaluation).

**Figure 4:**
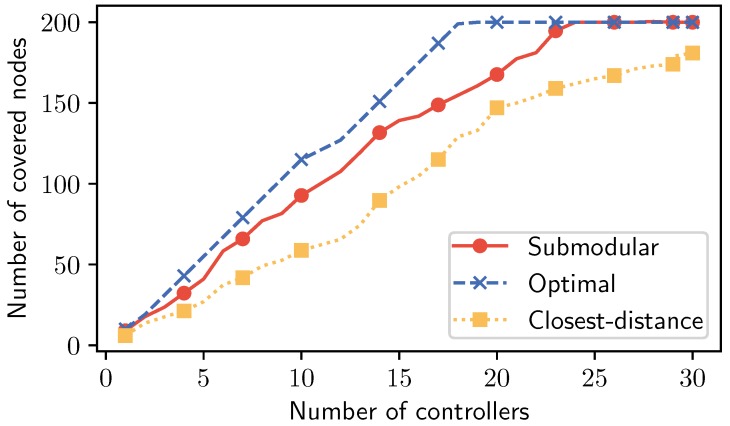
Impact of number of controllers on the controlled nodes.

**Impact of the number of controllers on the control nodes in the IoT network.** To evaluate the performance of our proposed algorithm, we measured the number of nodes controlled by the set of placement controllers. In the small setting (fewer than 50 nodes), these considered methods did not show a big difference in the comparison. However, there was a significant gap when the number of nodes was scaled up to 200, as shown in Figure 4. The Optimal baseline reached 19 controllers to control the system with 200 nodes. Following Optimal, Submodular could achieve a good performance with 23 controllers while Closest-distance could not satisfy this setting even with 30 controllers in the placement set.

**Figure 5:**
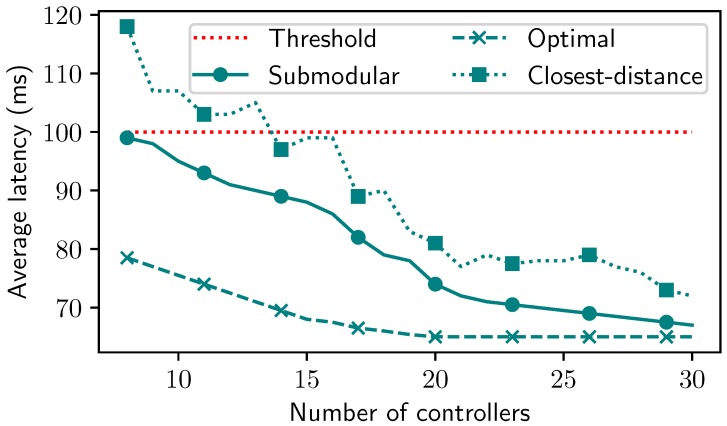
Average latency in different settings.

**Impact of the quality factor on the control nodes in the IoT network.** Following placement result in Figure 4, we conducted concretely the performance with the average latency aspect, as shown in Figure 5. To do this evaluation, we considered the setting with the latency threshold as 100 ms and found the controller set for 100 nodes. We then compared the methods in terms of the average latency of all methods. Closest-distance violated the threshold when the number of controllers was lower than 14. When we increased controllers in the placement set, the average latency of Closest-distance method reduced but its fluctuations remained since this aspect is not its objective. The Submodular method could obtain a promising result when its average latency was lower than the threshold level. Especially, the latency of Submodular approached closely to Optimal when the number of controllers reached 30 in our setting.

**Figure 6:**
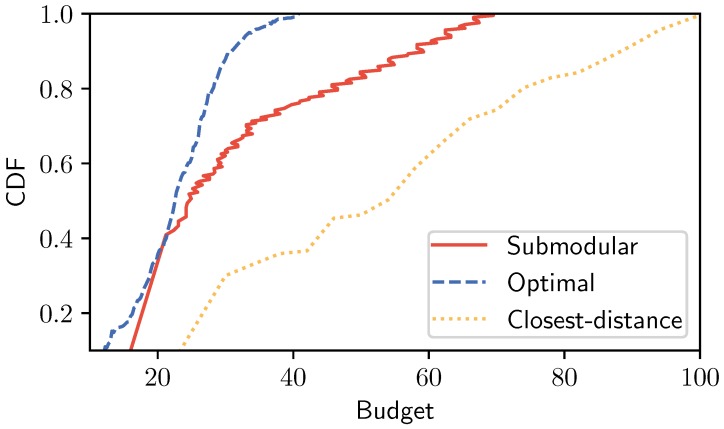
Impact of the budget setting on the performance.

**Impact of the budget constraint on the system performance.** In this case, we evaluated the system by considering the cumulative distribution function (CDF) result when increasing the budget constraint, as shown in Figure 6. With our setting, Submodular could fulfill the system (i.e., all controllers were assigned to be able to control every node in the system) when the system budget was set greater than 68, while Optimal baseline could satisfy the system with 40. Both methods had a big gap compared to the Closest-distance baseline when the budget constraint was set B>100. The reason Closest-distance baseline had a low performance is due to the assignment strategy based on the closest neighbor. In some cases, selected locations have more neighbor nodes than other candidates, but the placement cost might be very high.

## 5. Conclusions

In this paper, we focus on the controller placement problem for the distributed network, specifically for the IoT network, and apply the concept of submodularity to solve the class of controller placement problem, which is common in real systems. We present the proposed framework from the basic to the complicated controller placement problems under submodular theory. We explore different aspects of the controller placement problem under the submodular concept in order to emphasize that this is a promising framework to formulate and address various purposes of network providers. Corresponding to each formulation, we present a heuristic algorithm to address it based on the well-known Nemhauser algorithm to maximize the predefined submodular function. Through extensive simulation, we evaluated the performance and the efficiency of our model under different network settings. The evaluation demonstrated an outstanding result of our framework in terms of execution time, the number of controllers, and network latency.

Even though the submodular approach matches the controller placement problem, as aforementioned, in the perspective of the SDN controller in IoT networks, our framework does not consider concretely the relationship between controllers in the control channel, which is not independent in reality. To go deeper, a combination between the submodular theory and other methods to take into account the cost of the control plane will be the future target of our work.

## References

[1] Gil Herrera J.D.J., Vega J.F.B. (2016). Network functions virtualization: A survey. IEEE Lat. Am. Trans..

[2] Liyanage M., Gurtov A., Ylianttila M. (2015). Software Defined Mobile Networks (SDMN): Beyond LTE Network Architecture.

[3] Rivera A.R., Chin K.W., Soh S. (2015). Greco: An energy aware controller association algorithm for software defined networks. IEEE Commun. Lett..

[4] Wang G., Zhao Y., Huang J., Wang W. (2017). The controller placement problem in software defined networking: A survey. IEEE Network.

[5] Varghese B., Wang N., Barbhuiya S., Kilpatrick P., Nikolopoulos D.S. (2016). Challenges and opportunities in edge computing. arXiv.

[6] Heller B., Sherwood R., McKeown N. The controller placement problem. Proceedings of the First Workshop on Hot Topics in Software Defined Networks.

[7] Lovász L., Bachem A., Grötschel M., Korte B. (1983). Submodular functions and convexity. Mathematical Programming The State of the Art.

[8] Beheshti N., Zhang Y. Fast failover for control traffic in software-defined networks. Proceedings of the 2012 IEEE Global Communications Conference (GLOBECOM).

[9] Lange S., Gebert S., Zinner T., Tran-Gia P., Hock D., Jarschel M., Hoffmann M. (2015). Heuristic approaches to the controller placement problem in large scale sdn networks. IEEE Trans. Netw. Serv. Manag..

[10] Guo M., Bhattacharya P. Controller placement for improving resilience of software-defined networks. Proceedings of the 2013 Fourth International Conference on Networking and Distributed Computing (ICNDC).

[11] Ye X., Cheng G., Luo X. (2017). Maximizing sdn control resource utilization via switch migration. Comput. Netw..

[12] Dixit A., Hao F., Mukherjee S., Lakshman T.V., Kompella R.R. Elasticon an elastic distributed SDN controller. Proceedings of the 2014 ACM/IEEE Symposium on Architectures for Networking and Communications Systems (ANCS).

[13] Qin Q., Poularakis K., Iosifidis G., Tassiulas L. SDN controller placement at the edge: Optimizing delay and overheads. Proceedings of the IEEE INFOCOM 2018—IEEE Conference on Computer Communications.

[14] Killi B.P.R., Rao S.V. (2016). Optimal model for failure foresight capacitated controller placement in software-defined networks. IEEE Commun. Lett..

[15] Auroux S., Karl H. Efficient flow processing-aware controller placement in future wireless networks. Proceedings of the 2015 IEEE Wireless Communications and Networking Conference (WCNC).

[16] Gurobi. https://www.gurobi.com/.

[17] Cplex. https://www.ibm.com/analytics/cplex-optimizer.

[18] Nemhauser G.L., Wolsey L.A., Fisher M.L. (1978). An analysis of approximations for maximizing submodular set functions. Math. Program..

[19] Sviridenko M. (2004). A note on maximizing a submodular set function subject to a knapsack constraint. Oper. Res. Lett..

[20] Newman M.E. (2005). Power laws, pareto distributions and zipf’s law. Contemp. Phys..

